# Magnetically-Responsive Hydrogels for Modulation of Chondrogenic Commitment of Human Adipose-Derived Stem Cells

**DOI:** 10.3390/polym8020028

**Published:** 2016-01-25

**Authors:** Elena G. Popa, Vítor E. Santo, Márcia T. Rodrigues, Manuela E. Gomes

**Affiliations:** 1 3B’s Research Group—Biomaterials, Biodegradables and Biomimetics, University of Minho, Headquarters of the European Institute of Excellence on Tissue Engineering and Regenerative Medicine, AvePark, Taipas, Guimarães 4806-909, Portugal; getaolariu@gmail.com (E.G.P.); v.espirito.santo@itqb.unl.pt (V.E.S.); mrodrigues@dep.uminho.pt (M.T.R.); 2 ICVS/3B’s—PT Government Associate Laboratory, Braga/Guimarães, Portugal

**Keywords:** sulfated polysaccharides, magnetic nanoparticles, magnetic tissue engineering, stem cells, chondrogenic commitment, cartilage

## Abstract

Magnetic nanoparticles (MNPs) are attractive tools to overcome limitations of current regenerative medicine strategies, demonstrating potential to integrate therapeutic and diagnostic functionalities in highly controlled systems. In traditional tissue engineering (TE) approaches, the MNPs association with stem cells in a three-dimensional (3D) template offers the possibility to achieve a mechano-magnetic responsive system, enabling remote control actuation. Herein, we propose to study the role of MNPs integrated in κ-carrageenan (κC) hydrogels in the cellular response of human adipose-derived stem cells (hASCs) aiming at cartilage TE applications. The results indicated that the concentration of MNPs in the κC hydrogels influences cellular behavior, tuning a positive effect on cell viability, cell content and metabolic activity of hASCs, with the most promising outcomes found in 5% MNP-κC matrices. Although hASCs laden in MNPs-free- and MNPs-κC hydrogels showed similar metabolic and proliferation levels, MNPs κC hydrogels under magnetic actuation evidenced an instructive effect on hASCs, at a gene expression level, towards chondrogenic phenotype even in basic medium cultures. Therefore, the MNPs-based systems developed in this study may contribute to advanced strategies towards cartilage-like engineered substitutes.

## 1. Introduction

Magnetic nanoparticles (MNPs) have been investigated as an emergent nanotechnology tool for advanced biomedical applications due to the unique ability of MNPs to provide mechano-magnetic stimulus at a cellular level, and to remotely trigger and control MNPs actuation through an external magnetic field. MNPs have been applied in processes such as bioseparation [1], cell sorting [2], drug targeting [3] and gene delivery [4]. Furthermore, MNPs have been approved for clinical use as hyperthermia agents in cancer therapies [5], and as contrast agents in magnetic resonance imaging [6]. The applicability of magnetic fields and MNPs has been suggested towards controlled release of growth factors and bioactive molecules [7,8], and to stimulate cell embedding and cell patterning [9]. The potential of MNPs has also been demonstrated towards decreasing implant infection and increasing tissue growth [10]. Nevertheless, the impact of MNPs application in magnetic force-based tissue engineering (MagTE) approaches remains limited. The ability to manipulate and remotely control a new generation of multifunctional smart materials using MNPs envisions powerful guidance approaches with therapeutic effect through the modulation of cell motility, proliferation or differentiation [11]. Moreover, the application of MNPs in cell-based studies suggests a promising role of MNPs in magneto–mechanical stimulation of the cells, in particular to participate in the regulatory process of osteogenic and chondrogenic differentiation of stem cells by controlling the applied electromagnetic field [12,13,14]. Furthermore, the incorporation of MNPs in polymeric systems, for instance, in magnetic-responsive hydrogels may be useful in controlling cell guidance [15,16] and the *in vitro* angiogenesis process [17].

κ-Carrageenan (κC) matrices are interesting biomaterials to be used in MagTE strategies. These hydrogels have been previous studied by our research group [18,19], showing a suitable 3D environment to promote controlled release of growth factors (GFs) [20,21] and to support cell encapsulation for TE purposes, including cartilage-related applications, sustaining the viability and chondrogenic differentiation of several cell types [22,23]. The great potential of the κC-hydrogels for cartilage regeneration applications is due to the presence of sulfate groups, which mimic sulfated glycosaminoglycans (sGAG) naturally present in the cartilage extracellular matrix (ECM) [24,25]. Sulfated GAG potentiate the action of GFs through bond-specific interactions, while their degree of sulfation, molecular weight and structural composition has been described to modulate cellular response, including differentiation and gene expression [26,27]. κ-Carrageenan hydrogels combined with MNPs have been proposed as chemical stabilizers [28,29], as drug delivery systems with tailored release behavior [30,31], and also as magneto-elastic gels with magnetically tunable elasticity [32]. Therefore, the MagTE strategies involving κC matrices may enable the development of innovative solutions to significantly improve current available cartilage TE approaches through stimulation at the cellular level, with the advantage of having a leverage of a remote control with spatial and/or temporal precision.

Human adipose derived stem cells (hASCs) have also been reported for their differentiation ability towards the chondrogenic and osteogenic phenotypes [33,34] and specifically when embedded in κC hydrogels [35]. Thus, in the present study we have developed κC-based hydrogels with different concentrations of MNPs and evaluated the biological performance of hASCs laden in these hydrogels, namely cell viability, metabolic/proliferation profiles and chondrogenic potential for up to 21 days in chondrogenic and basal cultures, envisioning a cartilage engineering application.

## 2. Experimental Section

### 2.1. Development of MNPs-κC Hydrogels

κ-Carrageenan hydrogels were fabricated using κC (22048, Sigma, St. Louis, MO, USA) and potassium chloride (KCl, P5405, Sigma, St. Louis, MO, USA) solutions sterilized by heat (120 °C, 30 min). Commercial MNPs (45-00-252, Micromod, Rostock, Germany) were incorporated into the κC polymer solution at different concentrations, 2.5%, 5%, 10% (*v*/*v*). The MNPs selected for this study are iron oxide particles that include monodisperse magnetite aggregates with a diameter of about 250 nm and exhibit a plain surface. These particles are iron (II/III) oxide (Fe_3_O_4_, magnetite) (CAS: 1317-61-9) with a polydispersity index < 0.2. In our lab, authors verified that MNPs have a zeta potential of −11.6 mV. They are stable in aqueous buffers with pH > 4, and present a magnetization value of 46 emu/g iron (H = 1000 Oe), a saturation magnetization > 71 emu/g iron (H > 10,000 Oe) and a coercive field Hc of 0.481 kA/m. Following previous studies [27,35]; the κC hydrogels were prepared by pouring the κC solution with easily dispersed and colloidally stable MNPs into Petri dishes (55 mm Ø) followed by the gelation with KCl solution. Subsequently, hydrogel discs of Ø (5 ± 0.01) mm × (2.5 ± 0.46) mm height were obtained for cellular studies. MNPs-free-κC hydrogels were prepared and considered as reference materials.

### 2.2. Human Adipose Derived Stem Cells (hASCs): Isolation and Culture

Adipose surplus tissue samples were obtained upon informed consent from patients undergoing elective plastic surgery procedures under the scope of a previously established protocol with the Department of Plastic Surgery of the Hospital da Prelada (Porto, Portugal) and approved by the Hospital Ethical Committee In our lab, human ASCs have been routinely isolated by enzymatic digestion [36], and characterized for stemness potential by flow cytometry and RT-PCR for CD44, STRO-1, CD105 and CD90 markers, whose procedure is described in more detail elsewhere [37].

Briefly, the adipose tissue samples were digested with a solution of 0.2% collagenase type II (C6885, Sigma Aldrich, St. Louis, MO, USA) for 45 min at 37 °C under mild agitation. The digested tissue was filtered and centrifuged for 10 min. The resulting cell suspension was washed with lysis buffer to remove erythrocytes. Cells were resuspended in alpha Minimum Essential Medium (α-MEM, 12000-063 Gibco, Invitrogen, Waltham, MA, USA) with 10% Fetal Bovine Serum (FBS, 10270-106 Gibco, Invitrogen, Waltham, MA, USA; heat inactivated), 1% Antibiotic-Antimycotic (15240-062, Invitrogen, Waltham, MA, USA) and sodium bicarbonate (S5761, Sigma). Human ASCs were expanded until reaching a sufficient cell number for the encapsulation experiments. Cell culture medium was changed every 3 days and cells were used in passage one.

### 2.3. Encapsulation of hASCs in MNPs-κC Hydrogels

Human ASCs were detached by trypsin and encapsulated with a density of 2 × 10^6^ cells·mL^−1^ in MNPs-κC hydrogels. The MNPs solutions (with concentrations of 2.5%, 5% and 10% (*v*/*v*)) were blended with the cell suspension, and subsequently incorporated into the κC polymeric solution to a final polymer concentration of 1.75% (*w*/*v*). The MNPs-κC hydrogel laden with hASCs was then prepared by casting this mixture into sterile molds to form a solid gel at room temperature. The constructs made of MNPs-κC hydrogels laden with hASCs were cultured in basal medium for 1, 7, 14 and 21 days under static conditions without the actuation of an external magnetic stimulation.

To determine the magnetic responsiveness of hASCs as a modulatory factor in chondrogenic commitment, a magnetic stimulation of 350 mT applied to each construct was considered through an oscillation frequency of 2 Hz and 0.2 mm of displacement induced by a magnefect™ nano device (nanoTherics Ltd, Keele, UK). Human ASCs encapsulated in MNPs-κC hydrogels were also cultured in chondrogenic differentiation medium (CH) for 1, 7, 14 and 21 days, with media replaced every 3–4 days under constant magnetic stimulation. The CH was composed of Dulbecco’s Modified Eagle’s Medium-low glucose (DMEM, D5523, Sigma), supplemented with 10% FBS, 1% Antibiotic-Antimycotic, ITS + 1 Liquid Media Supplement (I2521, Sigma), 17 mM L-ascorbic acid (A4544, Sigma), 0.1 M sodium pyruvate (P4562, Sigma), 35 mM l-proline (P5607, Sigma), 1 mM dexamethasone (D4902, Sigma) and 10 ng/mL of human TGF-β1 (Transforming Growth Factor-β1, 14-8348, eBioscience, Barcelona, Spain).

### 2.4. Morphological Evaluation of MNPs-κC Hydrogels

The shape and surface characteristics of the developed hydrogels with encapsulated hASCs were examined in a stereomicroscope (Stemi 1000, Carl Zeiss Microscopy, LLC, New York, NY, USA) and pictures obtained using a camera PowerShot G6 (Canon Inc, Tokyo, Japan). Scanning Electron Microscope (SEM S-360, Leica, Cambridge, UK) was also used to characterize the morphology of the MNPs κC constructs. Samples selected for SEM analysis were washed in PBS, fixed in a solution of 2.5% glutaraldehyde (in PBS), dehydrated in a gradient series of ethanol solutions and allowed to dry at room temperature. Afterwards, the dehydrated templates were sputter coated with gold (Fisons Instruments, Sputter Coater SC502, England, UK).

### 2.5. Cell Viability and Metabolic Activity of hASCs in MNPs-κC Hydrogels

The viability of hASCs was assessed using Calcein AM and Propidium iodide (PI). For this purpose, a Calcein AM (1/1000, C3099, Invitrogen) solution was prepared in culture medium without phenol red and without FBS. PI was used at 1 mg/mL (P4170, Sigma) and DAPI (6-diamidino-2-phenylindole, 1 mg/mL, D9564, Sigma) was used for nuclei counterstaining. At each time point of the study, MNPs-κC hydrogels laden with cells were washed with PBS and incubated with the Calcein AM solution for 30 min at 37 °C. Subsequently, the samples were incubated with PI and DAPI fluorescent dyes for 10 and 20 min, respectively, and observed under a transmitted and reflected light microscope with apotome 2 (Axio Imager Z1m, Zeiss, Oberkochen, Germany). Images were acquired with an MRc3 camera and the AxioVision V.4.8 software (Zeiss).

The metabolic activity of hASCs in the MNPs-κC hydrogels was assessed by AlamarBlue^®^ Cell Viability Assay (AbD Serotec, Life technologies^®^, Oxford, UK) at 1, 7, 14 and 21 days of culture. A solution of Alamar Blue reagent 10% (*v*/*v*) was prepared and incubated with hASCs-MNPs κC hydrogels for 4 h at 37 °C and 5% CO_2_. Afterwards, supernatant was collected and the optical density (OD) read at 570 and 600 nm, using a microplate reader (Synergy HT, Bio-Tek Instruments, Winooski, VT, USA). Triplicates were made for each sample and experimental control. Culture medium with Alamar Blue reagent was used as negative control.

### 2.6. hASCs Proliferation in MNPs-κC Hydrogels

The proliferation of hASCs in MNPs-κC hydrogels was determined using a fluorimetric double strand DNA (dsDNA) quantification assay (P7589, PicoGreen^®^, Molecular Probes, Invitrogen). For this purpose, κC hydrogels laded with hASCs at 1, 7, 14 and 21 days post-culture were transferred into microtubes containing 1 mL of ultra-pure water, incubated for 1 h at 37 °C, and stored in a −80 °C freezer until use. Prior to dsDNA quantification, samples and experimental controls were thawed and sonicated for 15 min.

Samples, experimental controls and standards (ranging between 0 and 2 μg·mL^−1^) were mixed with a PicoGreen solution in 1:200 ratio. Triplicates were made for each sample, control and standard. The 96-well opaque white plate was incubated for 10 min in the dark and fluorescence was measured on a microplate ELISA reader (Synergy HT, Bio-Tek Instruments, Winooski, VT, USA) at 485/20 nm and 528/20 nm, respectively. A standard curve was created and dsDNA values of samples were read from the standard curve graph.

### 2.7. Chondrogenic Staining of hASCs Encapsulated in MNPs-κC Hydrogels

The cell laden MNPs-κC hydrogels and experimental controls were fixed with neutral buffered formalin solution (3.7% *v*/*v*) and stored at 4 °C until analysis. MNPs κC hydrogels were subjected to standard histological tissue processing for paraffin embedding (EC350-2, Microm, Thermo Scientific, Waltham, MA, USA). Sections with 3 μm thickness were obtained using a microtome (HM355S, Microm, Thermo scientific) and stained with a standard hematoxylin-eosin (H&E) (05-12011/L, 05-M10003, Bio-optica) protocol in an automatic stainer equipment (HMS740, Microm, Thermo Scientific).

Alcian blue staining was used to stain proteoglycans (PGs) and glycosaminoglycans (GAGs), naturally present in cartilage ECM. The staining was performed by rinsing the sections in acetic acid (3%, 151785, Sigma) followed by Alcian blue solution (1%) for 30 min, and by aqueous neutral red (861251, Sigma) for 1 min.

The sections were then dehydrated and mounted with Entellan^®^ (4111, Inopat) before being visualized under a transmitted and reflected light microscope with apotome 2 (Axio Imager Z1m, Zeiss, Oberkochen, Germany), and images acquired with an MRc5 camera and with the AxioVision V.4.8 software (Zeiss, Oberkochen, Germany).

### 2.8. Gene Expression of hASCs Laden in MNPs-κC Hydrogels

The total RNA from the MNPs-κC hydrogels laden with hASCs was extracted using TRI Reagent^®^ RNA Isolation Reagent (T9424, Sigma) accordingly to the technical datasheet specifications. RNA was quantified using a Nanodrop ND-1000 Spectrophotometer (Bonsai 06/2008 NanoDrop Technologies, Wilmington, DE, USA) and single strand complementary DNA was synthesized from 2 μg of RNA from both samples and experimental controls in a 40 μL reaction (qScript™ cDNA Synthesis Kit, Quanta Biosciences, Inc., Gaithersburg, MD, USA) using a MJ Mini™ Personal Thermal Cycler (Bio-Rad Laboratories, Berkeley, CA, USA) machine.

The relative gene expression of cartilage specific markers, whose primer sets are listed in Table 1, was assessed by real time polymerase chain reaction (rtPCR) using a SYBR Green PCR FastMix. The amount of transcription products of each marker was normalized to the average expression of the housekeeping gene: glyceraldehydes-3-phosphate-dehydrogenase (*GAPDH*) and calculated by the 2^−Δ*C*t^ method.

**Table 1:** Primers list of chondrogenic associated markers.

Target Gene	Primer Sequences (5′-3′)	*T*_m_ (°C)
*Sox9*	TACGACTACACCGACCACCA	58.4
	TTAGGATCATCTCGGCCATC	
*Collagen I*	CATCTCCCCTTCGTTTTTGA	55.3
	CCAAATCCGATGTTTCTGCT	
*Collagen II*	GACAATCTGGCTCCCAAC	56.4
	ACAGTCTTGCCCCACTTAC	
*GAPDH*	ACAGTCAGCCGCATCTTCTT	57.3
	ACGACCAAATCCGTTGACTC	

### 2.9. Statistical Analysis

Data resulted from Alamar Blue, dsDNA quantification and qRT-PCR analyses are given as mean ± standard deviation. Statistical analysis was carried out using GraphPad Prism 5.00 software (San Diego, CA, USA). Statistical differences were determined using one-way ANOVA or two-way ANOVA, followed by Bonferroni post-tests, to check the existence of statistical differences between sample groups.

## 3. Results

### 3.1. Morphological Evaluation of MNPs-κC Hydrogels

**Figure 1:**
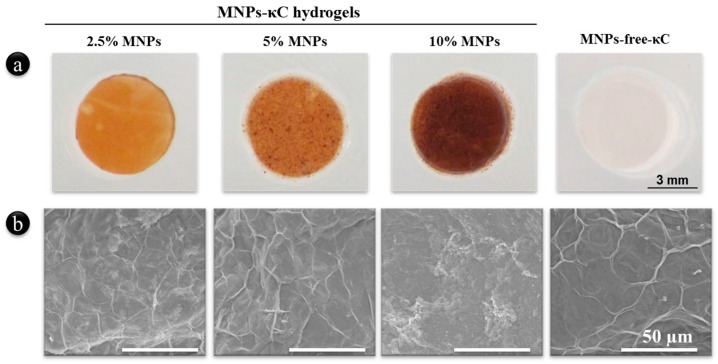
MNPs-κC hydrogels laden with hASCs in basal culture for 21 days. Macroscopic view of κC hydrogels loaded with different concentrations of MNPs, namely 2.5%, 5% and 10% (*v*/*v*), and the experimental control without MNPs, referred as MNPs-free-κC.

MNPs-κC hydrogels were successfully developed by *in situ* cross-linking with an ionic solution of KCl, as demonstrated in Figure 1. Since MNPs-free-κC hydrogels are colorless, the color intensity of the developed MNPs-κC hydrogels was related to the loading amounts of the iron oxide particles within the κC matrix (Figure 1).

The analysis of the surface morphology of freeze-dried MNPs-κC hydrogels at a microscopic level demonstrated that the increasing concentration of MNPs within the matrix did not seem to significantly impact the scaffold morphology in comparison with the MNP-free-κC hydrogels.

### 3.2. Influence of the MNPs Concentration on the Cell Morphology, Viability and Proliferation

**Figure 2:**
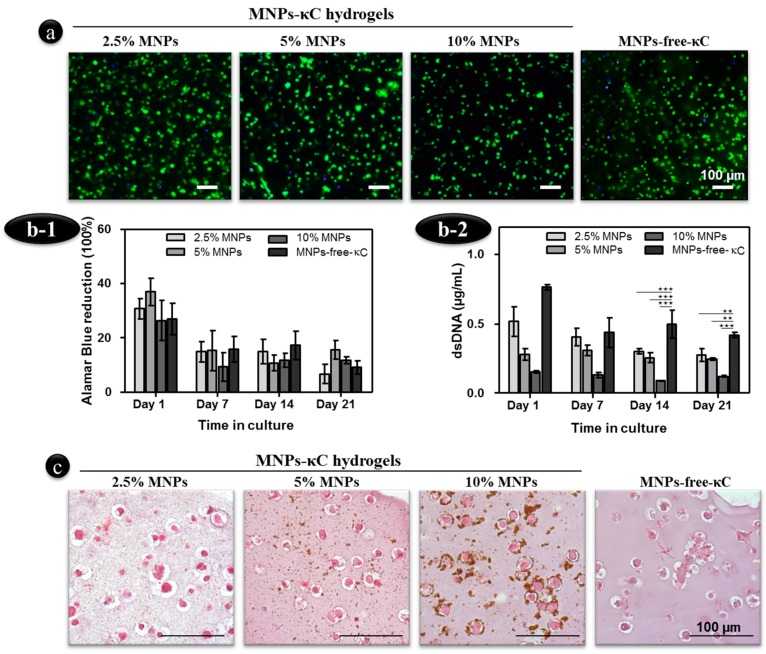
Influence of the MNPs concentration on the behavior of hASCs encapsulated in MNPs-κC hydrogels. (**a**) Live/dead assay of hASCs laden in MNPs-κC hydrogels cultured for 21 days. Cell nuclei were counterstained with DAPI (blue). Scale bar: 100 μm and original magnification of 5×; (**b-1**) Alamar Blue quantification did not reveal changes in the metabolic activity of hASCs cultured in MNPs- and MNPs-free-κC hydrogels but (**b-2**) dsDNA quantification of hASCs indicated statistically significant differences between MNPs- and MNPs-free-κC hydrogels at Day 14 and Day 21 (** *p* < 0.01; *** *p* < 0.001); (**c**) Light microscopy images of paraffin-embedded histological sections of MNPs- and MNPs-free-κC hydrogels with encapsulated hASCs stained with H&E and cultured in basal conditions for 21 days. Scale bar: 100 μm and original magnification of 20×.

The influence of MNPs concentration *per se*, that is without the trigger of an external magnetic field, was investigated over 21 days on hASCs-MNPs-κC hydrogels, as depicted in Figure 2.

Human ASCs viability cultured in MNPs-κC hydrogels (Figure 2a) was assessed by Live and Dead assay. Results show that the embedded hASCs displayed high levels of calcein AM staining and few dots of Propidium Iodide (PI) staining for all the κC hydrogels, demonstrating the physiological character of the developed hydrogels and that the addition of MNPs to the matrix did not significantly affect cell viability. Nevertheless, the κC hydrogels with 10% (*v*/*v*) MNPs presented the lowest number of viable cells among all studied conditions.

Human ASCs embedded in hydrogels with 5% (*v*/*v*) MNPs presented a more spread morphology within the κC matrix, as evidenced by their elongated fusiform morphology.

Interestingly, the presence of MNPs within the κC hydrogels did not significantly influence the metabolic activity of encapsulated hASCs, whose values were similar to MNPs-free hydrogel controls throughout the time in culture (Figure 2(b-1)).

In terms of cell content, a different trend was observed as MNPs-free-κC hydrogels presented statistically significant higher cell numbers throughout the culture period in comparison with MNPs-κC hydrogels (Figure 2(b-2)). The negative effect of the presence of MNPs dispersed in the hydrogel matrix was particularly pronounced for κC hydrogels with 10% (*v*/*v*) MNPs. This observation confirms the lower cell density observed for this formulation in the live/dead staining.

Altogether, by combining total metabolic activity and cell content data and despite the negative effect of the MNPs on cell proliferation, the cells dispersed in MNP-κC hydrogels present higher metabolic activity.

The increasing concentration of MNPs in the κC hydrogel was easily detected through the accumulation of dark MNPs spots in the histological cross-sections (Figure 2c). Changes in cell morphology in the presence of MNPs- and in MNPs-free hydrogels were also detected and monitored with hematoxylin-eosin (H&E) staining. hASCs in MNPs-κC hydrogels exhibit rounder-shape with individualized distribution within lacunae-like structures. Also, hASCs tend to be observed nearby the MNPs. This effect is more evident within the hydrogels with higher MNPs concentrations.

Based on the cellular response outcomes of MNPs-κC hydrogels, the 5% (*v*/*v*) MNPs formulation incorporated in κC hydrogels was selected to further investigate the effect of MNPs under the influence of an external magnetic field in promoting the chondrogenic commitment of hASCs laden in MNP-κC-hydrogels, as described in the following sections.

### 3.3. Reponse of hASCs Laden MNPs-κC Hydrogels under the Influence of an External Magnetic Stimulus

**Figure 3:**
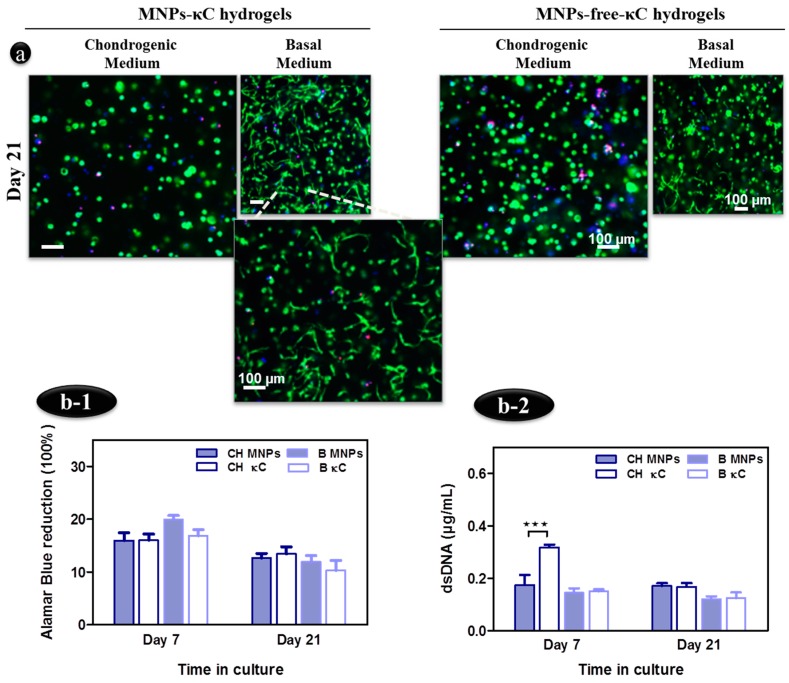
Assessment of hASCs viability and proliferation in MNPs-κC hydrogels under the actuation of an external magnetic field. (**a**) Live/dead assay of encapsulated hASCs in 5% (*v*/*v*) MNPs and MNPs-free-κC hydrogels after 21 days of culture. Scale bar: 100 μm and original magnification of 5×; (**b-1**) Metabolic activity assessed by Alamar Blue and (**b-2**) cell content assessed by dsDNA quantification. Differences between DNA content of hASCs on MNPs and MNPs-free-κC hydrogels were found to be statistically significant in the early time point (*** *p* < 0.001). MNPs- and MNPs-free-κC hydrogels with encapsulated hASCs (experimental controls) cultured in chondrogenic medium are designated CH MNPs and CH κC, respectively, while MNPs- and MNPs-free-κC hydrogels with encapsulated hASCs cultured in basal medium are labeled as B MNPs and B κC, respectively.

Figure 3a shows the viability levels of encapsulated hASCs in 5% (*v*/*v*) MNPs-κC hydrogels after 21 days in chondrogenic or basal medium, assessed by the Live/Dead assay. High cell viability was revealed by the calcein AM staining for all the conditions. Nevertheless, cell density tended to be slightly lower in MNPs-κC in comparison to MNPs-free-κC hydrogels. Regarding cell shape, hASCs embedded in hydrogels cultured in chondrogenic medium presented rounded-shape, whereas a more fusiform morphology of hASCs, likely related to cell spreading and migration phenomena, was detected in basal medium conditions (Figure 3a). Although the elongated and fusiform cell shape is predominantly observed in the presence of MNPs at 21 days of culture, it is also observed, to a lesser extent, in the MNPs-free-κC hydrogels (Figure 3a).

Moreover, the Alamar Blue assessment (Figure 3(b-1)) revealed that under the stimulation of a magnetic field, similar results were found for the metabolic activity of hASCs cultured in MNPs- and MNPs-free-κC hydrogels, over a period of 21 days. Additionally, cells exposed to MNPs triggered by an external magnetic field showed similar values of metabolic activity in chondrogenically induced- and basal-cultures along the time of culture (Figure 3(b-1)).

Although the DNA content of hASCs in MNPs-κC hydrogels was lower in chondrogenic cultures at the early time point, namely Day 7 (*** *p* < 0.001), in comparison to MNPs-free hydrogels, these registered differences were not significant (*p* > 0.05) after longer periods in culture (21 days) (Figure 3(b-2)).

Altogether, culture medium and the presence of MNPs dispersed in the hydrogel matrix did not significantly impact cell viability, metabolic activity and proliferation upon exposure to a magnetic field.

#### Assessment of hASCs Chondrogenic Commitment in MNPs-κC Hydrogels

**Figure 4:**
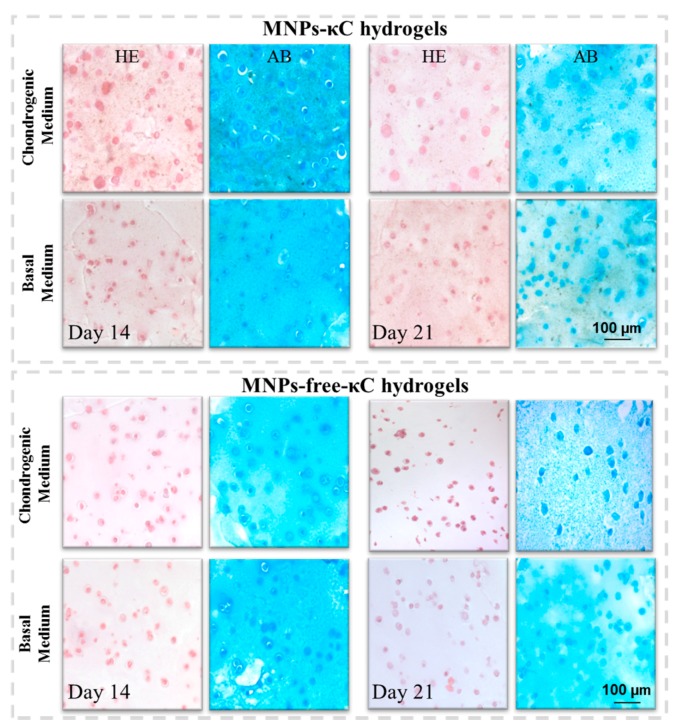
Light microscopy images of paraffin-embedded histological sections of hASCs laden in MNPs- and MNPs-free-κC hydrogels stained for hematoxylin-eosin (H&E) and Alcian Blue (AB). The MNPs were triggered by the actuation of an external magnetic field. Original magnification 20× and scale bar: 100 μm. MNPs-free-κC hydrogels with encapsulated hASCs are experimental controls.

H&E and AB staining of hASCs laden in MNPs-κC hydrogels under magnetic stimulation are represented in Figure 4. hASCs in H&E sections exhibited the typical chondrogenic-like round morphology in both MNPs- and MNPs-free-κC hydrogels independently of the culture medium. Furthermore, cells were homogenously distributed within the hydrogel matrix with an apparent increased cellular density in chondrogenic cultures.

The presence of round shaped cells within lacunae structures, often related to cartilaginous tissues, was more evident with AB staining, typically used for the detection of acidic sulfated proteoglycans (PGs). Although κC, a sulfated polysaccharide, was mildly stained with AB, the production of PGs by the encapsulated cells was identified by a darker and more intense stain nearby cells and lacunae-like structures. Furthermore, the blue stain observed in all conditions seems to be more intense after 14 days in MNPs-κC hydrogels. These findings suggest that biomolecules commonly found on the ECM of native cartilage were being produced and deposited in some extent in the hydrogel matrix.

**Figure 5:**
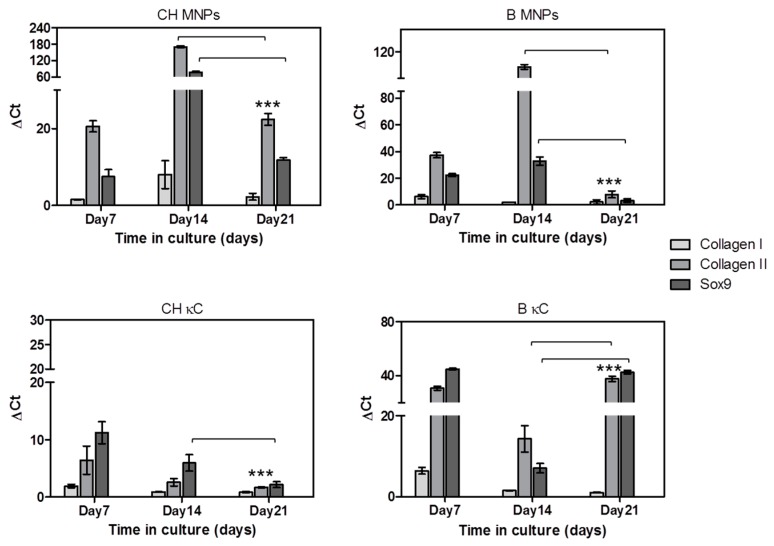
Relative expression of chondrogenic-specific transcripts, namely *Collagen type I*, *Collagen type II* and *Sox9* of hASCs cultured on MNPs-κC-hydrogels for 7, 14 and 21 days under the actuation of an external magnetic field. The expression of these genes was normalized against the housekeeping gene *GAPDH* and calculated by the ΔCT method. Data was analyzed by two-way ANOVA, followed by Bonferroni post-tests. Horizontal bracket lines denote statistically significant differences (*p* < 0.05) when comparing Day 14 with Day 21 time points and *** corresponds to a significant statistical difference (*p* < 0.001) for Day 21 in different conditions. MNPs- and MNPs-free-κC-hydrogels (experimental controls) cultured in chondrogenic medium are designated as CH MNPs and CH κC, respectively, while MNPs and MNPs-free-κC-hydrogels cultured in basal medium are labeled as B MNPs and B κC, respectively.

The quantification of the gene expression of chondrogenic associated markers was performed to ascertain hASCs chondrogenic commitment in MNPs-κC hydrogels cultured in chondrogenic and basal media. The relative expression of the chondrogenic genes was normalized against the housekeeping gene GAPDH and compared to hASCs cultured on Day 0 under the same conditions. The results of the gene expression of Collagen type I, Collagen type II and Sox9 are reported in Figure 5.

The expression of mRNA transcripts of Collagen I, Collagen II and Sox9 was up-regulated in all studied conditions during the time in culture (Figure 5). Overall, an increment pick on the expression of Collagen I, Collagen II (*p* < 0.05) and Sox9 (*p* < 0.05) was detected and was more pronounced by Day 14 in hASCs laden in MNPs-κC hydrogels, suggesting that the magnetic stimulation induced an earlier and stronger upregulation of chondrogenic-specific transcripts.

Moreover, the culture medium, namely chondrogenic supplemented medium, seems to have a role in the genetic expression of hASCs laden in MNPs-κC hydrogels, as the chondrogenic markers were highly expressed in comparison to basal medium conditions, independently of the time in culture.

## 4. Discussion

MNPs were successfully loaded into κC hydrogel matrices. Although κC is a high molecular weight macromolecule with charged sulfated groups, the stabilization of monodisperse magnetite was not affected. Therefore, the MNPs were easily dispersed and colloidally stable within the κC matrix, resulting in uniform distribution of MNPs in the κC solution, and afterwards in the developed κC hydrogels. Moreover, the presence of MNPs seems to increase the macroscopic stability of the developed hydrogels, as indicated by other studies [38,39].

The present findings also suggest that the concentration of MNPs in a κC matrix can tune the cellular response, and have a direct effect on cell viability, metabolic activity and proliferation of hASCs. Despite promising results with lower MNPs loads (2.5% and 5% *v*/*v*) into κC hydrogels, concentrations of 10% (*v*/*v*) MNPs led to a significant decrease on the proliferation levels of hASCs, being detrimental for cell colonization in the absence of magnetic stimulation. This is in agreement with previous reports showing that cell performance is dependent on MNPs dosage and also on the time course of MNPs–cell interaction [40,41].

The overall metabolic activity and DNA content maintenance over the culture period could be associated to the differentiation process of hASCs, as differentiating cells direct part of their energy to protein synthesis rather than cell division and proliferation [42]. In previous works [22,35], the authors have shown the potential of MNP-free-κC hydrogels to support hASCs differentiation towards the chondrogenic lineage. Human ASCs cultured in κC hydrogels showed long term viability and proliferation rates as well increased expression of chondrogenic associated markers.

Since hASCs are cultured in MNPs-κC hydrogels remotely triggered by an external magnetic field, the provided magnetic stimuli and cell–MNPs interactions may assist the process of chondrogenesis [43]. Human ASCs cultured in MNP-κC hydrogels showed evidences of chondrogenic commitment, namely the chondrogenic-like round cell morphology associated with lacunae-like formation, typical structures observed in cartilaginous tissues. The stronger detection of PGs within the ECM, and the higher genetic expression of chondrogenic-associated markers as collagen I, collagen II and Sox9, particularly after two weeks of culture, also sustain the commitment of hASCs towards the chondrogenic process.

Interestingly, under basal medium conditions, the inclusion of MNPs within the hydrogel matrix ensures the acquisition of a fusiform morphology by the hASCs that likely relates to the ability of these cells to spread within the κC matrix. Frequently, hydrogels lack adhesion points for the encapsulated cells to attach and colonize the hydrogel, and in this enriched construct, MNP- hydrogels might present additional adhesion points for the cells to attach and migrate. This also suggests that κC hydrogels have enough plasticity to allow cell movement and communication.

Although MNPs- and MNPs-free-κC hydrogels showed resembling metabolic and proliferation levels upon magnetic stimulation, specific differences were verified in stimulating the chondrogenic phenotype of laden hASCs. In the absence of MNPs, an increased expression of all chondrogenic-associated markers is observed in basal cultures. Such behavior is likely related to the application of a mechano-magnetic stimulus remotely provided by a magnetic field that seems to influence cell chondrogenic commitment even in the absence of MNPs in the hydrogel matrix. These results also suggest that cells respond to the magnetic field increasing the genetic expression of chondrogenic markers even without specific chondrogenic supplements added to the culture medium. Thus, in MNP-free-κC hydrogels, the magnetic actuation may have greater impact on chondrogenic differentiation than the biochemical factors present in the chondrogenic medium [44]. In general, hASC encapsulated in the MNPs-κC hydrogels showed higher expression values for all analyzed markers associated to the chondrogenic phenotype. This result is observed in both chondrogenic and basal medium cultures, suggesting that MNPs triggered by the actuation of an external magnetic field may have a synergistic effect on the chondrogenic commitment of hASCs laden in κC hydrogels [45].

Sox9 is highly expressed under chondrogenic supplementation, especially after 14 days in culture. Sox9 plays an essential role in determining chondrocyte fate and differentiation [46], and it is required for the gene expression of collagen type II and other cartilage-specific matrix proteins [47]. The enhanced expression of Sox9 suggests that the combination of MNP-κC hydrogel and magnetic stimulation plays a role in the regulation of the chondrogenic commitment of hASCs, especially in chondrogenic cultures. The increased Sox9 expression of stem cells when magnetic forces are applied is also confirmed by literature [48]. Furthermore, the qPCR results showed a low expression of collagen type I with a simultaneous high expression of collagen type II assessed for all experimental conditions. These results are particularly evident in MNPs κC hydrogels, with higher levels found in chondrogenic cultures. The high expression of collagen II detected mainly in MNPs-κC hydrogels confirms the presence of more mature chondrocytes after 14 days in culture, which also corresponds to the differentiation/maturation phase. The gene expression data is supported by the histological analysis, which demonstrated higher levels of newly synthesized ECM deposited within the hydrogel matrix at Day 14. Beyond Day 14, hASCs may be in a chondrocyte-like stage, supported by the fact that the expression pattern of all the markers is decreasing yet still up-regulated when compared to MNPs-free-κC hydrogels.

The obtained results suggest that the application of an external magnetic field in MNP-free hydrogels can influence biological mechanisms at the molecular level but the presence of MNP in κC hydrogels triggered by a magnetic field has a more significant effect in the expression of markers associated to the chondrogenic lineage. These markers were highly expressed by hASCs laden in MNPs-κC hydrogels, confirming that the external magnetic stimulation activates MNPs in κC hydrogels and assists the chondrogenic commitment of hASCs in a synergistic manner, as suggested by the literature [14,45,49].

Altogether, this work provides an important platform to understand, tailor and design new functional magnetic responsive constructs aiming at tissue engineering and regenerative medicine purposes. The incorporation of MNPs within a κC hydrogel matrix reveals benefits for assisting a cellular response of hASCs towards the chondrogenic lineage under constant exposure to an external magnetic field.

## 5. Conclusions

Magnetic responsive matrices are part of a new generation of smart biomimetic materials that provide remotely controlled stimulation, to better mimic and/or influence biological processes. A major advantage offered by MNPs-κC hydrogels is the ability to respond to stimuli provided by external magnetic forces towards the local guidance of biological processes on a targeted site, to be applied in regenerative medicine strategies. Moreover, the hydrogels developed in this study are able to support the biologic performance of hASCs towards the chondrogenic phenotype, modulated by the application of an external magnetic field even in basal medium conditions. It was also observed that the combination of biochemical (supplements to culture medium) and biophysical (magnetic MNPs activated by the external magnetic field) cues in a 3D environment seems to favor a synergistic effect on the commitment of hASCs towards chondrogenic phenotype in magnetic stimulated MNPs-κC constructs.

In summary, our results indicate that MNPs-κC hydrogels triggered by the application of a magnetic field constitute a versatile tool to modulate stem cell behavior and guide stem cell response towards the chondrogenic pathway by mimicking ECM properties and stimulating cell–matrix interactions.
